# Eight-year experience of maternal death surveillance in Morocco: qualitative study of stakeholders’ views at a subnational level

**DOI:** 10.1186/s12889-022-14556-0

**Published:** 2022-11-18

**Authors:** Saloua Abouchadi, Isabelle Godin, Wei-Hong Zhang, Vincent De Brouwere

**Affiliations:** 1 Ecole Nationale de Santé Publique (ENSP), Rabat, Morocco; 2 School of Public Health, Université Libre de Bruxelles (ULB), Brussels, Belgium; 3 Maternal and Reproductive Health Unit, Department of Public Health, Institute of Tropical Medicine (ITM), Antwerp, Belgium; 4 Department of Public Health and Primary Care, Faculty of Medicine and Health Sciences, Ghent University, Ghent, Belgium

**Keywords:** Maternal death, Surveillance system, Implementation, Evaluation, Use, Sustainability, Facilitators, Barriers, Stakeholders, Morocco

## Abstract

**Background:**

Since 2009, Morocco has been implementing the Maternal Death Surveillance System (MDSS). The results obtained indicate significant regional variations in terms of implementation stage, completeness of maternal death reporting, and information use for action. The objective of this research is to better understand the contextual factors involved in the implementation process and use of MDSS, with a focus on the facilitators and barriers, as experienced by stakeholders in health regions.

**Methods:**

Evaluation research was conducted in 2017 based on a descriptive qualitative study using semi-structured in-depth interviews, in four out of the twelve health regions of Morocco. A total of thirty-one in-depth interviews were held with members of regional committees of maternal death reviews (RC-MDR) and other key informant staff. Interviews focused on participants’ views and their experiences with the MDSS since the introduction in 2009. We conducted thematic analysis relied on inductive and deductive approaches. Applying the Consolidated Framework for Implementation Research guided data analysis and reporting findings.

**Findings:**

Engaging leadership at all health system levels, regular training of district and regional MDSS coordinators and supportive supervision at a national level were the most important MDSS implementation facilitators. Reported barriers were essentially related to the review system: Irregular review meetings, blame culture, high turn-over of RC-MDR members, lack of analytical capacity to inform the review process and formulate recommendations, finally limited accountability for recommendation follow-up. While financial incentives boosted MDSS adoption, they were nonetheless a substantial barrier to its sustainability.

**Conclusions:**

The MDSS is a complex process that requires taking numerous steps, including the commitment of multiple stakeholders with varying roles as well as information sharing across health system levels. Contextual factors that influence MDSS implementation at the sub-national level are to be considered. Horizontal and vertical communication about MDSS goals and feedback is crucial to strengthen stakeholders’ commitment, hence improving quality and use of MDSS. Furthermore, health regions should place emphasis on making high-quality recommendations in partnerships between the regional management teams, RC-MDR members and external stakeholders.

**Supplementary Information:**

The online version contains supplementary material available at 10.1186/s12889-022-14556-0.

## Contributions to literature

• Barriers and facilitators of MDSS implementation were deeply analyzed at a subnational level using the Consolidated Framework for Implementation Research (CFIR).
• The research highlighted stakeholders’ requirement for implementing MDSS, And their subjective experiences with sustaining such a system
• Ethical and legal issues should be anticipated to ensure effective and sustainable MDSS implementation.
• The study indicates that the main factors enabling MDSS implementation include MOH support, skilled and trained staff, communication regarding MDSS goals and feedback and sub national stakeholders’ early involvement in sustainability discussions.

## Background

As part of the maternal mortality reduction strategy, low and middle-income countries (LMICs) have been working to establish comprehensive Maternal Death Surveillance and Response (MDSR) systems since 2013. However, glaring gaps exist between the development of the national MDSR policies and their in-country implementation [1, 2]. Operationalize maternal death review committees and implementing actions in response to recommendations are a main shortcoming in this system [2]. In 2017, LMICs were urged to include perinatal deaths (MPDSR) in their surveillance systems, which added to the challenges [3, 4].

For more than a decade, Morocco had no prevalent harmonized or standardized process for examining and studying maternal deaths. This depended on individual health facilities performing clinical audits (audit research studies or pilot projects funded by the Ministry of Health (MOH) and its partners). Following their apparent success, the MOH called for scaling up voluntary clinical audits in obstetrics in March 2001. However, few maternity hospitals began audits and even fewer have continued them [5–8].

With the announcement of the health policy for 2008–2012 and the increasing national focus on accelerating maternal mortality reduction, the MOH sought to operationalize a National Maternal Death Surveillance System (MDSS) [9]. In 2008, the MOH mandated the completion of notification forms for every death of women of reproductive age (WRA). It instructed health regions and districts to adhere to the national MDSS guidelines, issued in 2009, based on training provided to all district and regional MDSS coordinators [9]. The Minister of Health appointed a national experts committee (NEC) on confidential inquiries into maternal deaths (CIMD) and RC-MDR. Three national CIMDs (2009, 2010 and 2015) have been conducted to date, and all emphasized that most deaths could be prevented if proper actions are taken in health care facilities [10, 11].

Nevertheless, it is important to examine the weaknesses in the current MDSS. In 2017, the MDSS was still operational in ten of the twelve regions. In seven regions, all MDSS components were implemented. Only four regions successfully integrated MDSS activities into routine practices [11]. Significant under-reporting, lack of investigation and review of maternal deaths were barriers to a well-established national system [1, 11, 12].

For such a public health surveillance system to have a true impact, stakeholder commitment throughout the surveillance process is necessary. Stakeholders can help interpret the reported data based on their knowledge of the health outcome or the environment where the data is collected. They may also respond to the information generated by the surveillance system and recommend or influence the surveillance system evaluation to ensure that it meets its goals [13].

Stakeholders and their relationships are at the heart of the implementation process, as are implementation climates and communication channels [14]. Yet, there is a lack of structured research based on stakeholders’ needs in building MPDSR and their own subjective experiences related to sustaining it [14].

The purpose of this study is to understand stakeholders’ views at the regional health level related to facilitators and barriers of MDSS implementation and its use between 2009 and 2017. It is Morocco’s first qualitative MDSS evaluation with the aim of generating evidence-based recommendations to strengthen the MDSS. The findings can thereby guide policymakers in Morocco and other LMICs, and help plans and adjustments to make this system more useful at the subnational level.

## Methodology

### Research design

We adopted a descriptive qualitative research design [15, 16] to describe and explore facilitators and barriers to MDSS implementation in health regions, as well as to make evidence-based recommendations for MDSS improvement.

Such descriptive qualitative research is relevant for summarizing and understanding a topic by recognizing its subjective nature as well as the viewpoints of those involved [15, 17]. The focus on producing rich descriptions of a phenomenon from experienced people offers a unique opportunity to get inside knowledge and learn from how they perceive their context [18, 19]. The findings closely reflect the initial research question and are of particular interest to practitioners and policymakers [15, 20].

Because they build on prior exploratory work, descriptive qualitative studies may be utilized to conduct a more focused investigation. They may, however, generate unexpected results when a phenomenon is explored in new contexts [16]. This flexibility allows for adjustments in research focus and methods as empirical findings emerge. The concepts or phenomena studied can guide decision-making throughout the research process, allowing the most relevant methods to answer the research question [16].

### Study setting and recruitment

Considering the nationwide implementation of MDSS, we selected four regions using purposive sampling among the ten regions that demonstrated evidence of maternal death surveillance practice in 2017. An additional Excel file shows this in more detail (see Additional file 1). To maximize data heterogeneity, we examined MDSS implementation level and monitoring MDSS indicators as well as population’s vulnerability. The characteristics of the study regions selected are described in Table 1.

**Table 1:** Region’s characteristics

Region	**R (A)**	**R (B)**	**R (C)**	**R (D)**	**National**	**Source**
Area (km^2^)	40 423	28 374	19 448	53 789	710 850	1
Population	4 236 892	2 520 776	7 122 876	2 676 847	33 848 242	
Urban population % (min. – max)^a^	60,5 (13,0–98,2)	49,1 (18,2–69,7)	73,6 (18,9–100)	56,1 (29,5–94,9)	60,3 (34,3–93,4)	
WRA (2016)	1 173 795	687 696	1 876 257	761 555	9 318 000	2
Number of births per year (2016)	82 902	52 615	126 400	51 658	674 227	
% Births attended by skilled health personnel (2016)	84,8	76,6	94,3	86,8	86,6	3
% C—section	24,3	15,7	23,1	25,7	21,1	
% Antenatal care coverage—at least four visits (2016)	53,7	37,1	59,4	54,6	53,5	
Number of maternal deaths (2016)	38	25	24	36	298	4
MDSS implementation scoring (0 – 30 points)	7,9	23,9	17,5	24,4	14,9	5

1. High Commission for Planning (HCP), Morocco. https://www.hcp.ma/links/Les-sites-des-Directions-regionales-et-Provinciales-du-HCP_ai61143.html

2. Health statistics 2016, Service des Etudes et de l’Information Sanitaire (SEIS), MOH, Morocco. https://www.sante.gov.ma/Documents/2019/11/Sant%C3%A9%20en%20chiffres%202016.pdf

3. Morocco DHS, 2018. https://www.sante.gov.ma/Documents/2020/03/Rapport%20ENPSF% 202,018% 202i % C3%A8me%20%C3%A9dition.pdf

4. MDSS 2017, Health regions

5. Abouchadi S. Le système de surveillance des décès maternels au Maroc: Pour optimiser la prise de décision au niveau des régions [Dissertation on internet]. Brussels: Université Libre de Bruxelles (ULB); 2022. http://hdl.handle.net/2013/ULB-DIPOT:oai:dipot.ulb.ac.be:2013/ 341,974

^a^Variances in urban population between districts within the same region

The regional MDSS coordinators (RCo) supplied a list of stakeholders involved in MDSS management and/or conducting MDRs (RC-MDR members). We defined stakeholders at the health region level as those individuals who contribute to the maternal death surveillance and/or review process, use the results obtained, or have influence due to their position over the MDSS’s implementation and sustainability [13, 21]. The stakeholders include district and regional maternal health managers (tasked with data collection and/or MDRs), practitioners who are members of the RC-MDR and district or regional public health officials. Among forty-seven potential participants, we could not find contact information for eight health workers due to retirement, departure for private practice, or transfer to another region. Thirty-nine were invited all to take part in the scheduled interviews with facilitation from RCo. All agreed in principle to attend, except for one anesthesiologist-resuscitator (AN-RE), who declined the invitation and no longer wanted to be a RC-MDR member. The principal investigator (PI) met with the interviewees at the health region’s office. She called the ten people who did not show up that day to schedule another appointment. Three participants were interviewed via phone: a regional health director (RHD), an obstetrician-gynecologist (OB-GYN), and a head of the regional health observatory (RHO). Even after constant callbacks, seven participants were unable to attend: One RHD, three OB-GYNs, one of whom left for the private sector, two AN-REs, and one midwife.

### Data collection and reflexivity

The data collection method chosen was in-depth interviews (IDIs). This method is particularly suitable for letting the respondent express him/herself in his/her own words, to be able to bring out original research material [22].

**Fig. 1:**
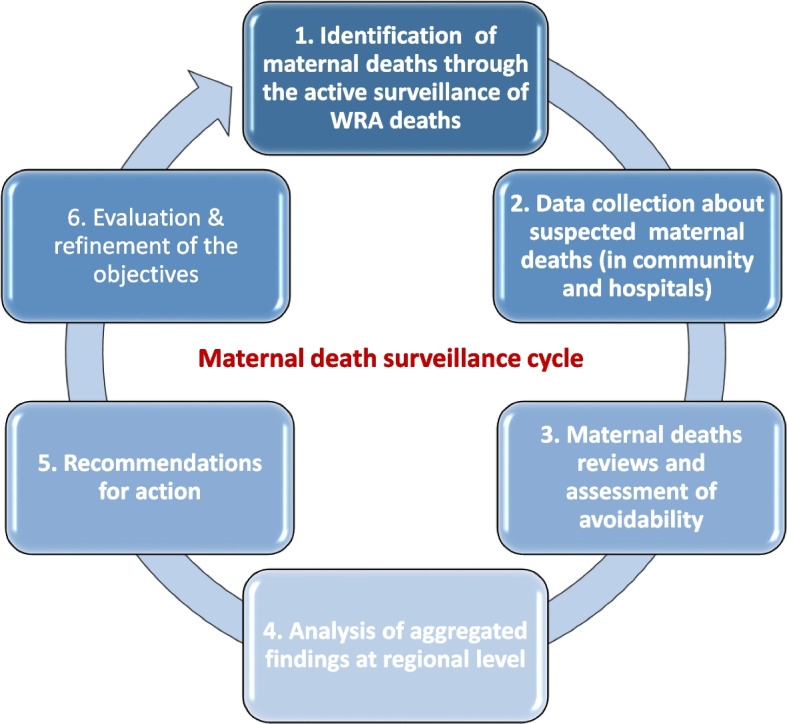
Maternal death surveillance cycle

To further understand the MDSS implementation process, a topic semi-structured guide was prepared after reviewing the national MDSS guidelines. Steps of the maternal death surveillance cycle were all covered (Fig. 1).

The topic guide addressed the following key themes: How was the MDSS initiated in the region? How were participants initiated and introduced to their roles? How was maternal death notified and investigate? How were MDR meetings held, and aggregate data analyzed? How were recommendations formulated and implemented?

The interview guide focused on barriers and facilitators of MDSS implementation and use. However, due to the IDI’s interactive nature, questions could be changed or added considering data obtained from earlier interviews [22]. The full topic guide can be found in Additional file 2.

The PI (SA) collected data between May 2017 and August 2017. She led IDIs in person at the health region office in a private place, depending on respondent’s availability and willingness to participate. Interviews were conducted in French and tape-recorded with the participants’ verbal consent. The interview was concluded once no added information regarding MDSS seemed to emerge, and the interviewer had a full understanding of the participant’s perspective. Only four stakeholders did not give their verbal consent to be recorded. In this case, the interviewer took detailed notes during the discussion and reconstructed them shortly after completing the interview.

Researchers also reviewed additional documentation when available (i.e., MDSS action plans, monitoring indicators, RC-MDR meeting notes, regional reports on CIMD) to complete interviews and acquire an in-depth understanding of participants’ key statements.

### Data analysis

Two student midwives transcribed recorded interviews into French in Microsoft text format. The PI (SA) cross-examined all transcriptions for consistency and accuracy before she imported them to N VIVO 11 Starter for data management.

The exploitation of the data was that of thematic analysis using inductive and deductive approaches. In 2006, Braun and Clarke clarified the conceptual basis and practical aspects of thematic analysis in the field of psychology [23]. Although various authors were already making extensive use of it in the field of sociology. The aim was to generate new knowledge from inductive reasoning, reasoning that consists of moving from the specific to the general. The mix of an inductive and deductive qualitative approach combines the advantages of inductive reasoning: generating new knowledge and consolidating it with a theoretical framework, as Pearse states: “deductive qualitative research, the theoretical propositions derived from a review of the literature serve as its departure point, informing how the data is collected” [24]. As stated by Casula and al, to encompass a deductive approach to inductive may increase the scientific rigor, while allowing new theory or new hypotheses to emerge [25].

Two authors (SA and VDB) separately coded first inductively the facilitators and barriers influencing MDSS implementation. Then, they deductively mapped them to the CFIR domains and constructs. Data reliability was confirmed by comparing the encoding produced independently. Agreements and discrepancies were documented, and any divergence was resolved by consensus, through discussion within the research team.

The theoretical framework for research was adapted from the Consolidated Framework for Implementation Research (CFIR) [26–29]. Thirty-nine CFIR constructs are organized into five domains: (1) intervention characteristics, (2) outer setting, (3) inner setting, (4) individual characteristics, and (5) process [28]. The CFIR constructs by domain along with a brief description are listed in Additional file 3.

**Fig. 2:**
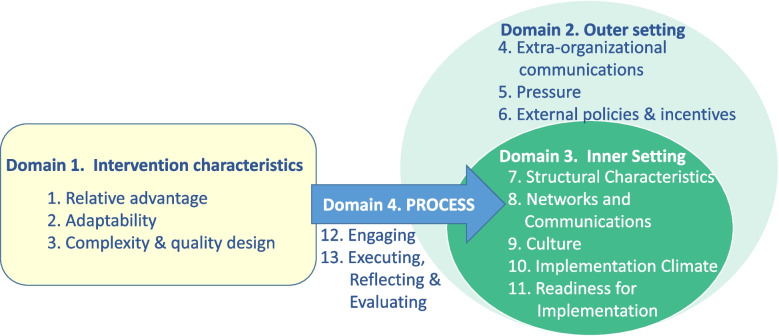
Conceptual framework for MDSS implementation adapted from CFIR [28]

Domain 4 (individual characteristics) was not applied because our analysis unit was the health region rather than the individual. A codebook was developed and refined to describe the constructs selected, those rejected or merged, as well as the underlying justification (See Additional file 4). Figure 2 illustrates the conceptual framework used to guide data coding, data analysis and findings reporting.

The adapted CFIR contained four domains (intervention characteristics, outer setting, inner setting, and process) and fourteen constructs. The MDSS is a multifaceted intervention with six interacting components. Its characteristics (relative advantage, adaptability, complexity, and quality design) may influence implementation in a health region (inner setting). The healthcare system is hierarchically organized with interrelationships between health regions and the national level. Changes in the outer setting (extra organizational communications, political and hierarchical pressure, and external policies and incentives) can have an impact on implementation, often mediated through changes in the inner setting (structural characteristics, networks and communications, culture, implementation climate, and readiness for implementation). Successful implementation usually requires achieving individual and organizational level use of the intervention as designed. Figure 2 represents the implementation process by an arrow that symbolizes the engaging, executing, reflecting, and evaluating implementation, aimed at the direction of the inner setting for an effective implementation.

Results were presented in narrative format following the CFIR Framework stages. We determined what qualified as a theme when a similar finding came from different respondent profiles and when it could shed interesting light on the research question and thus offer an angle of analysis for our results. The tree node structure developed for thematic analysis is summarized in Additional file 5. Verbatim that best illustrated the significant points shared by most respondents were selected and translated into English by the PI (SA). Co-authors discussed the translation, which has been revised by a professional translator. The Consolidated criteria for Reporting Qualitative research (COREQ) (See Additional file 6) were used to structure reporting methods and the findings [30].

## Results

### Participants

A total of 31 participants were interviewed: 14 women and 17 men. They were aged from 30 to 60 years old (median 46) and had an average of five-year experience with MDSS (min: 1, max: 8). The interviews lasted 67 min on average, although they ranged from 20 to 120 min (Table 2).

**Table 2:** Characteristics of the participants

Characteristics (*N* = 31)		N
Age (years)	*30 – 40*	8
	*40 – 50*	15
	*50 – 60*	8
Gender	*M*	17
	*F*	14
Number of participants by health region	*R-(A)*	8
	*R-(B)*	9
	*R-(C)*	5
	*R-(D)*	9
Length of service (Number of years)	< *10*	4
	*10 – 15*	5
	*15 – 20*	8
	≥ *20*	14
Function	*Regional health director (RHD)*^a^	3
	*Hospital director*	1
	*District health director (DHD)*^b^	1
	*Head of public health and epidemiological surveillance service (PHESS)*	4
	*Head of the regional health observatory (RHO)*	4
	*Head of health care service (HCS)*	1
	*Maternal health program manager*	8
	*Healthcare provider*	7
	*Health facility network service (HFNS) manager*^c^	*2*
Professional profile	*OB-GYN*	4
	*Midwife*	6
	*General practitioner (GP)*	9
	*Nurse*	3
	*Pediatrician*	1
	*Public health specialist*	6
	*Statistician*	1
	*System and network engineer*	1
Involvement in MDSS (Number of years)	*1 – 3*	6
	*3 – 6*	8
	*6 – 8*	17
Role in MDSS	*Regional MDSS coordinator*^d^ *(RCo)*	5
	*District MDSS coordinator*^e^*(DCo)*	5
	*Member of the RC-MDR*^f^	13
	*Member of the Regional Task Force (RTF)*^g^	9

^a^The RHD is the top-level manager in charge of determining and implementing health and social protection policies in his/her region

^b^DHD is responsible for deciding and implementing an action plan designed for health and social protection of the population of the district

^c^HFNS is a district-level department in charge of planning and implementing public health programs at the local level

^d^The RCo is officially appointed to monitor MDSS activities in the health region and serve as the RC-MDR secretary. He or she is usually the maternal health program manager who is attached to the public health surveillance service

^e^The DCo is designated for collecting data on maternal deaths at the district level

^f^RC-MDR is a multidisciplinary committee in the health region that performs reviews and identifies contributing factors to maternal deaths. It is representative of OB-GYNs, midwives, AN-REs, GP, and public health. Based on analysis and interpretation of aggregated findings from reviews, the RC-MDR develop recommendations and writes the regional confidential enquiry report to be shared amongst stakeholders involved in maternal health

^g^RTF is a steering group established in 2017 to implement regional action plans designed to eliminate preventable deaths among mothers, newborns, and children under the age of five. It is managed by the RHD and is composed of district and health region managers (DHD, hospital directors, HFNS managers, Head of PHESS, etc.), expert members as well as MOH officials

### Facilitators and barriers to MDSS implementation

Findings are based on four investigated CFIR domains: (i) Intervention characteristics, (ii) Outer Setting, (iii) Inner Setting, (iv) and Process. Accordingly, we present respondents’ understanding of facilitators and barriers to initiating and sustaining MDSS, as well as suggestions for future improvement.

### Intervention characteristics

The first MDSS characteristics domain includes three constructs: relative advantage, adaptability, and complexity and quality design.

#### Relative advantage

The MOH developed and implemented the MDSS in 2009. Almost all interviewed stakeholders agreed that the MDSS was better than the status quo or clinical audit. The most significant MDSS advantages were increased awareness of maternal mortality issues among health personnel, improved understanding of maternal deaths and families’ experiences, and enhanced communication between health facilities and districts. The MDSS also contributed to improve working conditions and reduce intra-hospital maternal mortality.

*- “This system has truly benefited our facilities. The hospital manager has upgraded the surgery room. Regional reviews were quite valuable. For example, there was no death this year (2017) in the aftermath of nine deaths in 2016.” (An OB-GYN–M–3 years (Y) of MDSS experience).*

#### Adaptability

Allowing districts and health regions to adjust the MDSS to local constraints was identified as a facilitator for implementation. In some districts, the DCo performed preliminary inquiries and verbal autopsies before families left the hospital, despite the allowed 6-week limit set by national guidelines. In all regions, RC-MDR now meets once or twice a year instead of quarterly.

*- “Municipalities report women’s deaths to the health districts, but not to the primary healthcare facilities ... I have to perform preliminary investigations and verbal autopsies at the hospital, because no one else will.” (A DCo–F–7 Y).*

#### Complexity and quality design

Stakeholders perceived the MDSS’s quality design and complexity as implementation barriers. Data collection was time-consuming and labor-intensive due to difficulties in identifying maternal deaths and filling out excessively long questionnaires. The lack of a high-performance IT application was to blame for delays in data aggregation and analysis across districts.

- “The system is complex, and the questionnaires are lengthy. The MOH must revise and reduce them to strictly the most important variables.” (A RCo–M–8 Y).

### Outer setting

This domain considers factors which are external to the health region: political and hierarchy pressure, national policy and incentives, and extra organizational communication.

#### Political and hierarchy pressure

From 2008 to 2012, there emerged a strong government determination to reduce the maternal mortality, which was an essential facilitator of on-the-ground implementation. All stakeholders involved in MDSS during this period were convinced that the national political commitment and pressure from top Health Ministry Officials inspires regional and local “leadership commitment”.

- “We always follow the Minister’s strategy. Human resources and maternal mortality are the two top priorities.” (A RHD–M–6 Y).

#### National policy and incentives

In 2009, 2011 and 2015–2016, the MOH provided MDSS training for DCo and RCo, with support of development partners such as WHO, UNFPA and UNICEF. Financial incentives were reserved to DCo and professionals in primary healthcare facilities to fund costs related to data collection on WRA and pregnancy-related deaths. The same applied to RC-MDR members traveling to the regional health directorate to review maternal death records. However, these incentives are provided as part of projects involving partners, which implies that they were not given to all districts on a regular basis. While financial motivation was acknowledged as an implementation facilitator, stakeholders stressed the negative effect of the one-off reward on MDSS sustainability and integration into routine practice.

Several stakeholders also raised concerns about failure to implement the overall healthcare system development policy. As a result, women do not receive prompt life-saving healthcare, which is critical to the MDSS's relevance and, hence, its sustainability.

*- “Maternity wards meeting standards and other planned interventions as part of maternal mortality reduction action plan (2008-2012) were foiled. Nothing materialized in practical! The MDSS is one part of the strategy to reduce maternal mortality. It cannot be developed on a standalone basis; it is part of a full package! ” (A DHD–M–6 Y).*

#### Extra organizational communication

The appointment of a national coordinator at the MOH level facilitated intensive communication and strong networking with DCo and RCo during early MDSS implementation stages.

*- “ In 2009, the MOH ensured that MDSS adoption was closely monitored. DCo have frequent telephone conversation and email communication with the national MDSS coordinator. Districts and regions are now more connected with one another.” (A regional maternal health program manager–F–7 Y).*

Informal channels of communication improve collaboration with external actors, particularly local authorities, and thus WRA death reporting. However, because of the risk of penalties for health practitioners, no feedback is provided to territorial collectivities.

*- “ We organize meetings to make local authorities aware of certain issues, but we cannot share the full report with them. It is a double-edged sword. They would focus on the report statement that “95% of deaths are avoidable”. Therefore, fault is attributed to the healthcare system and health professionals.” (A RCo–M–8 Y).*

### Inner setting

The third domain consisted of five constructs referring to factors that were internal to the health region: structural characteristics, internal communications, cultural environment, implementation climate, and readiness for implementation.

#### Structural characteristics

Most stakeholders mentioned staffing shortages as a major barrier to MDSS implementation, especially those related to applying recommendations. Respondents in one region with low staff turnover reported an important level of workplace camaraderie, and teamwork positively influenced MDSS implementation and sustainability.

*- “Most of the personnel come from neighboring areas. We have been working together for a long time. We are knowledgeable about the regional specificities. This familiarity makes work easier.” (A HFNS manager–M–8 Y).*

#### Internal communications

The important formal and informal communication links between health regions and districts are influencing MDSS implementation and sustainability and cannot be overstated. Regular health managers’ meetings at distinct levels of the health system were considered as implementation facilitators, just as the absence of these meetings was viewed as a barrier by hospital medical and nursing personnel.

*- “It is simply a matter of lack of communication. They inform us of a meeting on the same day or the day before. So, I am no longer able to attend most of these meetings because I cannot arrange for them on such short notice. Moreover, we do not receive a copy of the regional report in advance.” (An OB-GYN–M–4 Y).*

#### Cultural environment

The blame culture was a major source of concern and still is today in public health services. Health workers feel afraid of maternal deaths when managers refer to them as murders. Midwives are unable to speak openly about causes and circumstances of maternal deaths.

*- “ Every time a woman dies, we are afraid of being punished. The head nurse was turning people against us. Doctors were trying to blame us. This is no longer the case; the current hierarchy, as well as doctors support us.” (A Head nurse of a maternity hospital–F–3 Y).*

The RC-MDR meetings were not open and constructive, and members sought to find the culprit. Health professionals’ defensive attitude made it difficult to accept the questioning of practices. The external experts’ (university hospital professors) presence in meetings was valuable in that regard.

*- “It is very important to have an external view to guarantee transparent and beneficial reviews, especially when the hospital staff has a defensive attitude...” (A GYN-OB–M–4 Y).*

#### Implementation climate

While some argue for MDSS incompatibility with the urgent need for immediate maternal death information because they face immense pressure from policymakers, the community, and the media, all stakeholders recognize that MDSS is an added workload. They experience difficulties incorporating MDSS activities into their workflow and information system.

*- “ We are adding new processes, therefore the personnel must work harder… We need to combine efforts and use a single data collection form. Teams lack time! “ (A DHD–M–6 Y).*

The relative priority, i.e., the shared perception of the importance of the implementation within the health region, had a detrimental effect on MDSS sustainability. At the start, national strategic and political priorities drove the “push” towards adopting the MDSS. When the MOH introduced other interventions, a lower level of priority was given to MDSS.

*- “ We spent our days in 2016 and the first quarter of 2017, collecting data from health programs to update the national health information system. We also had to prepare the regional healthcare provision plan.” (A RCo–F–8 Y).*

The lack of internal organizational incentives and rewards was a strong barrier to MDSS integration and sustainability. As health workers saw MDSS as requiring extra efforts, they expected some form of compensation.

*- “ Due to a staffing shortage in one district, environmental health coordinators agreed to perform preliminary home inquiries into WRA deaths rather than primary healthcare workers doing it. Initially, the MOH offered a cash incentive. But, when such a payment was removed, they refused to complete the task since it was outside of their scope of work.” (A RCo–F–8 Y).*

All stakeholders involved in the MDSS implementation were not aware of the MDSS goals. Managers were more informed than healthcare providers. They prioritize counting of maternal deaths over MDRs and recommendation implementation.

*- “ The problem is not just that maternal deaths are under-reported in comparison to expectations. We must also discuss what we did in response to the identified maternal deaths.” (An OB-GYN–F–7Y).*

Feedback was given through regional coordination and task force meetings. Several interviewees expressed disappointment regarding the time spent at these meetings on reflection on the issue and collective evaluation of the MDSS. This limited the opportunity for organizational learning based on MDSS data.

- “What task force? They did not comment on the regional report! This kind of committee only complicates matters.” (A RCo–F–6 Y).

#### Readiness for implementation

Engaged leadership, access to adequate time, financial and material resources, as well as access to knowledge and information supported the initial stages of MDSS implementation.

- “Our partner gave us around 3,000 euros in 2015. It was enough to meet expenses. MDSS benefited from this financial support.” (A RCo–F–8 Y).

The lack of additional resources for MDSS was a barrier to sustainability. A minimum amount of funds is required to cover field investigators’ travel expenses, organize RC-MDR meetings, analyze data, organize training, and edit the regional report.

*- “We meet in an unpleasant setting, a small room. We hold meetings in the afternoons and go beyond 4:30 pm. I have nothing to offer RC-MDR members except chocolate that I bought myself. They are pleasant people with whom I get along well. So, they agree to coming back anyway” (A RCo–F–8 Y).*

On one hand, almost all DCo and RCo agreed that successive training sessions (in 2009, 2011 and 2015–2016) helped in dissemination of information and knowledge. The continuous implementation support from the national MDSS coordinator participated in keeping them informed and building momentum. On the other hand, health professionals do not appear to have the same level of knowledge and information. Several RC-MDR members have expressed an interest in MDR and data analysis training. They also sought guidelines for reporting, presenting, and wording of recommendations.

*- “ I worked as a ward midwife in 2009... At the HFNS meeting, only the hospital’s maternity ward team leaders were present. Midwives were unaware of MDSS. When I was appointed to manage the maternal health program at HFNS in 2013, they handed me the MDSS guidelines. Then, in 2015, I attended the DCo’s training.” (A DCo–F–4 Y).*

### Implementation process

The implementation process was considered crucial in terms of engaging relevant individuals and executing and evaluating the MDSS.

#### Engaging

Several respondents emphasized the importance of formally appointing key stakeholders, particularly RCo, DCo and RC-MDR members. They recognized the RCo as an essential actor based on the backing of hierarchy, and his/her relationships with district teams and external actors. RCo were all trained and had worked, except for one, on MDSS for a long time (at least six years). However, when the MDSS depends on a single person, continuity is no longer ensured if she/he leaves.

*- “The stability of the RCo implies that he has a wide range of interpersonal relationships. He benefits from hierarchy’s support, regional personnel’s trust, and a well-developed network.” (A HFNS manager–M–8 Y).*

The maternal health program manager and the HFNS manager have a substantial effect on the success of MDSS implementation at the district level. Training, regular monitoring via quarterly reports, supportive supervision, coordination meetings, and other strategies, such as mentorship, are all needed to maintain commitment.

*- “The district maternal health program manager and the head of HFNS are key players of the MDSS at the district level. Those trained in 2009 and 2011 were committed. Others who came afterwards were not. They invented excuses: "I am not trained". The training in 2016 helped revitalize MDSS in districts.” (A RCo-F–1 Y).*

Many RC-MDR members saw their involvement in MDSS as theoretical. They stated how the RCo just approached them by phone to join the RC-MRD. They were discouraged when not invited to meetings in timely manner, when they did not receive the report for review or were not asked to provide feedback prior to the task force meeting. Some interviewees questioned the members’ selection process and underlined the role of university hospital academics in ensuring an objective review of deaths at regional meetings.

*- “RD-MDR members should be unanimously chosen with the support of their colleagues. Furthermore, due to recurring confrontations, the presence at the meeting of individuals from outside the region, with scientific credentials such as a university hospital senior professor, is preferred.” (A RHD–M–6 Y).*

#### Executing, reflecting, and evaluating

The level of MDSS implementation varied within and across the four regions, but all demonstrated evidence of practice. Both WRA and maternal deaths were under-reported according to DCo and RCo. The preliminary inquiries to identify suspected maternal deaths were not systematic. Fear of maternal complications or blame for a maternal death led to unnecessary referrals to the next level of care, increasing the workload on the referral hospital.

According to managers, RC-MDR members blamed deaths on factors that were frequently beyond their control, such as lack of prenatal care, community challenges, and infrastructural concerns. Although hierarchy contested those conclusions, RC-MDR members were frustrated that their recommendations had not been adopted.

*- “What frustrates me is that after all these years, I still do not see any added value due to my work nor any progress to justify my sacrifices. The committee detects flaws and suggests feasible solutions that we are unable to implement. So, year after year, the same problems and death causes reoccur.” (A RCo–F-8 Y).*

## Discussion

This study provides an in-depth qualitative evaluation of the MDSS implementation process using the CFIR as a theoretical lens. Potential facilitators of MDSS implementation included the intervention’s adaptability and its relative advantage compared to current practices (intervention characteristics), political and hierarchy pressure and communication with the national level (outer setting), access to knowledge and information (inner setting), and RCo and DCo level of commitment (process). Potential barriers included perception of the intervention as complex (intervention characteristics), not always compatible with current workflows and a prevalent blame culture (inner setting), competing priorities of the MOH (outer setting), weak RC-MDR and insufficient support for implementing recommendations (process).

### A complex surveillance system

Political commitment and hierarchy pressure were the main reasons for the early MDSS adoption in less than a year. In several LMICs, the “top-down approach” backed by strong political and administrative buy-in and national leadership enabled the MDSR implementation [31–34]. However, both top-down and bottom-up approaches are required to ensure successful implementation and sustainability [31–33, 35].

Building a sustainable MDSS was difficult in the four regions of our study due to its complexity, concurrent political priorities, and lack of resources. Complexity arises from two MDSS components that are interrelated. The district surveillance and MDRs are combined to inform regional and national CIMD. The review system, which is the responsibility of the health region, should be kept separate from the district’s surveillance system. Districts must prioritize the use of medical certificates of death cause, particularly in hospitals and communes served by health offices. Because more than half of pregnancy-related deaths occurred in intensive care units, operating theaters, maternity wards, and emergency services, physicians and nurses working in these settings should be targeted [12].

Maternal death surveillance is time-consuming at the district level because of the lengthy data collection tool. The questionnaire covers detailed medical information about the woman and her death, which data is collected through hospital records, staff interviews, and/or verbal autopsies involving family members. This information is essential for the RC-MDR to determine whether the death was caused by avoidable factors. Furthermore, once the file is de-identified at the district level and centralized in the region, it is no longer possible to obtain additional information from DCo. This issue must be addressed: How to balance competing demands for simplicity, quality, and exhaustivity when the district does not directly use the collected data? A brief questionnaire might be integrated into daily work and timely completed with other specific forms, depending on regional priorities. Since 2015, a web-based system is in place in seven regions. However, it is not yet fully functioning. Countries with well-established MDR systems use technology to facilitate data analysis, allowing for faster aggregation and analysis of data sourced from districts or states [2, 36, 37].

The ability to maintain trained staff on surveillance and MDRs was a concern recognized by all health regions. Getting funding to keep training and supplies going was challenging. This highlights the importance of regions launching local programs of training, including web-based training, to address issues such as under-trained staff and high staff turnover. Continuous technical skill development planning is required, as is a mechanism to assess stakeholders’ knowledge level [38, 39].

### Overcoming blame culture

Lack of confidentiality and blame culture continue to be big issues for efficiently engaging stakeholders in MDRs, sharing MDSS results, and implementing recommendations. The use of a systems-theoretical approach to MDRs should help identify causal factors at all levels of the system, learning from mistakes, and designing system-level changes to avoid these concerns in the future [40]. Despite consensus that the “no blame” model is far more successful in making health care facilities safer for women giving birth, managers have yet embraced the model. A punitive malpractice regime still dominates the error landscape [8, 41–44]. The healthcare system should strive towards a culture that accepts human errors as part of human nature and encourages people to discuss them without fear of repercussions. Achieving the right balance between “no blame” and accountability is challenging. Debate should address rather than a “no blame” culture, a “just culture,” which distinguishes between blameworthy and blameless conducts [45, 46]. Women’s safety requires ongoing efforts to improve practices, training, information technology, and culture throughout the hospital, not only in the maternity ward. Professional norms that see patient harm as a social problem should guide efforts. Solutions can be found if healthcare workers and managers work together [46–48].

### Buy-in by health professionals and external actors

Our study revealed that most healthcare providers did not recognize MDSS benefits in their work, MDSS being too slow to produce practical results. They struggle to distinguish between the regional CIMD and the clinical audit approach. The lack of feedback from RC-MDR and NEC regarding MDSS performance hinders the implementation process, highlighting the benefit of establishing feedback and communication mechanisms. Both healthcare providers and managers are frustrated because community, medias and policymakers do not appreciate efforts to reduce maternal deaths. The MOH should encourage regions to share maternal death surveillance findings at international and national conferences and meetings, to increase awareness and understanding of maternal deaths occurring locally. Regional communication that includes external stakeholders is crucial for supporting motivation and building a learning culture. This suggests the importance of managing expectations and finding ways to engage with district and regional staff.

Incentives were crucial factors in both outer and inner settings. In 2009 and 2010, the MOH provided technical and financial incentives to DCo in all districts to encourage them to collect data for MDSS. In 2015, only six regions got onetime cash incentives. This short-lived support was not sufficient to ensure MDSS sustainability, which should lead to consideration and discussion early in the planning process of MDSS feasibility and sustainability and the need to engage effectively field actors. Morocco’s political pressure implied a rapid MDSS launch and a large-scale implementation. The MOH is responsible for maintaining programs such as MDSS that meet the government’s public health priorities. Difficulty in monitoring MDSS performance and implementing a proper incentive mechanism for data collection and MDRs is still one of the system’s most serious sustainability challenges.

### Putting recommendations into practice

The CIMD makes recommendations based on elaborate procedures. Unfortunately, the MDSS dissemination and response component appear to be underdeveloped. Stakeholders are confident in MDSS usefulness, yet the lack of implementing recommendations is discouraging. Region's health officials expressed doubts related to MDRs’ reliability and criticized the recommendation-making process. The current monitoring system focuses on the completeness of maternal death reporting. No indicators track progress in implementing recommendations. Although each region conducted an annual evaluation, the format differed, with some producing an overall summary report and others summarizing information in a slide presentation. Few health regions developed strategies for implementing recommendations and no systematic follow‐up of previous recommendations was organized.

In numerous countries, a large gap exists between data production and the ability to translate that data into usable information, formulate appropriate recommendations, and subsequently initiate relevant public health actions [44, 49]. In South Africa, each triennial report included recommendations. However, no specific indicators, targets, or timelines were defined, and no tracking system was used [43]. Several studies specifically mention the challenge of not having funds to execute MDR recommendations [14].

## Strengths and limitations of the study

Our study is the first qualitative study in Morocco, using an implementation framework to analyze factors influencing MDSS implementation and use. The CFIR was used to guide data coding, analysis, and reporting of findings. Each decision and rationale for selecting and reporting of CFIR constructs is documented. Since the stakeholders interviewed were from diverse levels (health region, district and health facility), the qualitative data was more comprehensive. A check by a research member contributed to reduce the bias in description and interpretation of IDIs. We also reviewed MDSS documentation from 2009 to 2017 in each region.

Nevertheless, our study has some limitations. First, to make the interview guide more understandable to the respondents, the CFIR was not used explicitly. Hence, some CFIR constructs may be missing information.

Second, we limited the IDIs to healthcare professionals who were RC-MDR members. External stakeholders and other healthcare professionals offering maternal healthcare services should translate results and recommendations into quality improvement actions. However, due to a lack of research resources and challenges in reaching out to these people, it was impossible to include them in our study. This is not a significant limitation, as various participants reported previous MDSS experiences while they were not RC-MDR members.

Another potential limitation is related to memory bias because we conducted the study between 2017 and 2018. However, the fact that the PI conducted the interviews herself, supervised and verified the transcripts, and took detailed field notes all contributed to reducing the memory bias.

Finally, the PI was the national MDSS coordinator between 2009 and 2013, then head of the national unit of maternal death surveillance when she conducted the research. This position had no influence on the selection of study participants. According to inclusion criteria, RCo compiled the list of names of potential participants, not the PI. Furthermore, as national coordinator, she mostly communicated with RCo, with whom she had a good relationship, based on trust and respect. This connection was extremely helpful in conducting the study. For most participants, the interviews were their first contact with the PI.

## Conclusion

The MDSS is a complex process that utilizes a continuous cycle from identifying, reporting, and reviewing deaths to actions to improve healthcare services and avoid future deaths. This system has yielded three national reports on maternal deaths and only five regional reports. MDSS data and those reports are either unavailable or difficult to acquire. This shows that the appropriation of the MDSS, even 10 years after its launch is still a problem. Much effort is still required to produce high-quality routine analyses.

The MOH also planned to launch a similar neonatal deaths surveillance system in 2009. Despite repeated attempt, it has not been done because neonatal deaths are more frequent and more difficult to investigate. Our study is the only one to explore MDSS implementation factors in Morocco. Given our results, the MOH should carefully consider whether to adopt the MPDSR approach and combine perinatal and MDRs at the health facility level, or to first aim to improve the existing system.

Whatever approach is adopted, some important key issues must be addressed: MOH support, skilled and trained staff, horizontal and vertical communication related to MDSS goals and feedback and early involvement of subnational stakeholders in sustainability discussions.

## Supplementary Information

**Additional file 1.** Assessment of health region level MDSS in Morocco. **Figure S1.** Score by health region for each level of implementation. **Figure S2.** Implementation progress on the schematic scoring scale.

**Additional file 2.** In-depth interview guide.

**Additional file 3.** Dimensions and constructs of the CFIR and their description.

**Additional file 4.** CFIR Codebook adapted to MDSS.

**Additional file 5.** Summary of the node tree developed for thematic analysis (NVivo Starter 11).

**Additional file 6.** COREQ 32 item Checklist.

**Additional file 7.** Grey literature.

## Data Availability

All relevant data are available within the paper and its Supporting Information files.

## Abbreviations

AN-RE: Anesthesiologist-Resuscitator
COREQ: Consolidated criteria for Reporting Qualitative research
CFIR: Consolidated Framework for Implementation Research
CIMD: Confidential inquiries into maternal deaths
DCo: District MDSS coordinator
DHD: District Health director
DHS: Demographic and Health Survey
F: Female
HFNS: Healthcare facility network service
IDI: In-depth interview
LMICs: Low and middle-income countries
M: Male
MDSS: Maternal Death Surveillance System
MDR(s): Maternal death review(s)
M(P)DSR: Maternal (Perinatal) Death Surveillance and Response
MOH: Ministry of Health
NEC: National experts committee
OB-GYN: Obstetrician-gynecologist
PHESS: Public health and epidemiological surveillance service
PI: Principal investigator
RC: Regional committee
RCo: Regional MDSS coordinator
RHD: Regional health director
WRA: Women of reproductive age
