# Supplementary material for: Eight-year experience of maternal death surveillance in Morocco: qualitative study of stakeholders’ views at a subnational level
Source: BMC Public Health. 2022 Nov 18;22:2111. doi: 10.1186/s12889-022-14556-0 (PMC9673401; doi:10.1186/s12889-022-14556-0)
Supplement: Supplementary file 2 — Additional file 2. In-depth interview guide. [file 12889_2022_14556_MOESM2_ESM.pdf]

Additional file 2: In-depth interview guide *(original version in French)*

Date : /___/___/_______/

Start time: /_____h : _____min/ End time : /_____h : _____min/

Region: /_____________________/

Demographic questions	
1.	Gender (Male/Female)
2.	Age of participant (in years)
3.	Occupation of participant
4.	Seniority at work (years or months)
5.	How long have you been working in the region?
6.	Where did you work before?

(Questions for regional coordinators of MDSS)

Themes	Questions
**MDSS management**	How long have you been MDSS Regional coordinator?
	What are your activities as MDSS Regional coordinator?
	Who helps you with these activities?
	Did you receive any training on MDSS? how many times?
	Please tell me how satisfied you were and the weakest and strongest aspects of the training in your opinion?
	What data of MDSS are available in your region? (From 2009 to 2017)
**Notification and Investigation of deaths among WRA**	Please describe step-by-step procedures of notifying and investigating deaths among WRA in the region?
	What are the constraints on notifying and investigating deaths among WRA in the region?
**In-depth investigation of pregnancy-related deaths**	Please describe step-by-step procedures of investigating pregnancy-related deaths in the region?
	What are the constraints on investigating pregnancy-related deaths in the region?
**Maternal deaths reviews (MDRs)**	How does the RC-MDRs work in your region?
	What are the procedures and deadlines for transmitting maternal deaths files from districts to the region?
	How does a MDRs session is conducting in practice?
	What are your constraints on reviewing pregnancy-related deaths?
	How would you describe the quality of maternal deaths files transmitted by districts?
**Data analysis**	Please describe step-by-step procedures of MDSS data analysis at the health regional level?
	What were the findings of the maternal deaths data analysis in 2016?
	What are your constraints on analyzing data at regional level?
**Annual regional report**	How do you prepare your annual regional report on the confidential enquiry into maternal deaths?
	How do you disseminate the results of the regional report?
**Implementation of recommendations**	What will become of the recommendations resulting from the MDSS?
**Additional information on MDSS**	How do you assess the MDSS coverage/efficiency in your region?
	How many people are involved in the MDSS? (Districts and region)
	How often do you have fieldwork supervisions for MDSS? (Per year, per district?)
	How do you determine the necessary financial needs to sustain the MDSS in your region?
	What sources of funding are available for covering the costs of this system?
	What is the total budget allocated per year to MDSS?
**Exit questions**	Please tell me about your overall experience with MDSS?
	What are the most common obstacles you personally face for following up on this system?
	What are your suggestions on how we can improve MDSS at the region level?
	What are your expectations from the MOH and/or the Health Regional Directorate to be able to do your best work as a coordinator?

(Questions for other stakeholders)

Themes	Questions
**MDSS implementation**	Can you please tell me when and how the MDSS was introduced in your region?
	What do you know about the MDSS?
	When and how did you get involved in the MDSS?
	What are your responsibilities as part of the MDSS?
	Have you ever received any training about MDSS? If yes, from where/whom? Was it useful for you?
	How is organized in practice the maternal deaths surveillance in your region?
	How do you assess the MDSS implementation in your region?
	Please tell me about your overall experience with the MDSS implementation process? (Whether here or in another region)
	In your opinion, what are issues that need to be addressed in the future for the sustainability of the MDSS in your region?
	What are your suggestions for improving maternal mortality surveillance at the regional level?
**Use of results of MDSS**	What is the place of the MDSS within the regional strategy to reduce maternal mortality?
	What mechanisms did you set up to disseminate the recommendations resulting from the MDSS (regional report / RC-MDRs)?
	How do you use the MDSS analysis results and recommendations in your region?
	What are the circumstances/situations/reasons why MDSS data are used?
	Can you explain why MDSS findings are used (or not) in regional decision-making?
	What do you suggest transforming the MDSS into an effective regional tool for action?
	What are the needs of the region to improve the MDSS use and how to address them?
**Exit questions**	Please tell me about your overall experience with MDSS?
	What are the most common obstacles you personally face to achieve your role in this system?
	What are your suggestions on how we can improve MDSS at the region level?
	What are your expectations from the MOH and/or the Health Regional Directorate to be able to do your best work as a RC-MDRs member?
