# Supplementary material for: Eight-year experience of maternal death surveillance in Morocco: qualitative study of stakeholders’ views at a subnational level
Source: BMC Public Health. 2022 Nov 18;22:2111. doi: 10.1186/s12889-022-14556-0 (PMC9673401; doi:10.1186/s12889-022-14556-0)
Supplement: Supplementary file 4 — Additional file 4. CFIR Codebook adapted to MDSS. [file 12889_2022_14556_MOESM4_ESM.pdf]

Additional file 4: CFIR Codebook adapted to MDSS.

CFIR is intended to be flexible in application in ways that researchers can tailor it to the specific intervention design, factors and context being studied. The domain 4 (individual characteristics) was not applied in our study because our unit of analysis was the health region rather than the individual. We removed the CFIR codes that were not relevant for our study and / or the national context and merged some themes due to the overlap of the information in practice. In more details, we removed intervention source, evidence strength and quality, trialability (ability to test the intervention on a small scale) and cost. We merged design quality and complexity from Intervention characteristics domain. We removed needs and resources of those served by the organization from Outer setting domain and in the Process domain. Finally, we removed planning and merged execution, reflection and evaluation into one construct.

	Construct and Short Description	Coding criteria	Justification of unsuccessful constructs
**I. INTERVENTION CHARACTERISTICS**			
A	Intervention Source:<br>Perception of key stakeholders about whether the MDSS is externally or internally developed.	--	National context: "top-down" approach to design health interventions; The MDSS has been implemented eight years ago; No verbatims expressed any perception regarding neither centrality of the decision (as problematic) to implement MDSS, nor the evidence strength and quality. Most of the interviewers tried to remember the date or mention the national strategy to reduce maternal mortality including MDSS. These two constructs were non-relevant in our study and overlapped with another construct: Access to Knowledge & Information (Readiness for implementation).
B	Evidence Strength & Quality:<br>Stakeholders' perceptions of the quality and validity of evidence supporting the belief that the MDSS will have desired outcomes.	--	
**C**	**Relative Advantage:**<br>Stakeholders' perception of advantage to implementing the MDSS.	Inclusion Criteria: Statements that demonstrate MDSS benefits, compared to the previous situation (No MDSS), or that it is better (or worse) than past or existing programs (e.g other clinical audit methods or another surveillance system).<br>Exclusion Criteria: Statements that do or do not demonstrate a strong need for the MDSS and/or that maternal death is an untenable public health problem, e.g. statements that the MDSS is absolutely necessary or that it is redundant with other programs (code to **Tension for Change**).	
**D**	**Adaptability:**<br>Degree to which the MDSS can be adapted, tailored, refined, or reinvented to meet local needs.	Inclusion Criteria: Statements regarding the flexibility and (in)ability to adapt and change the MDSS to regional/provincial context, to operating conditions, and information needs, to depending on workflows of individual organizations (e.g., complaints about the rigidity of processes, variations in implementation observed across subnational levels, suggestions for MDSS improvement).<br>Exclusion Criteria: Statements that the MDSS did or did not need to be adapted to **Compatibility**. It may be possible to infer that a large number of suggestions for improvement demonstrates a lack of compatibility.	
E	Trialability:<br>Ability to test the intervention on a small scale in the organization, and to be able to reverse course (undo implementation) if warranted.	--	No pilot sites<br>A National roll-out of the MDSS in 2009
**G**	**Design Quality and complexity:**<br>Perceived excellence in how the MDSS is designed and difficulty of MDSS itself that refers to both its structure and difficulty of its operation.	Inclusion Criteria: Statements regarding features that make the MDSS use easy or complex (e.g., clear case definition and data flow, difficulty in completing surveillance data, time to complete surveillance data and reviews, web-based system that facilitates data capture and transfers surveillance data to the aggregate level).<br>Exclusion Criteria: Statements regarding the **complexity of implementation**, are coded to other appropriate CFIR codes (**Available Resources** or **Engaging**).	Complexity was merged with Design quality because concepts overlapped
H	Cost:<br>MDSS costs, including investment, supply, and opportunity costs.	--	Not relevant for the study because costs are covered by the MOH. MDSS' regional coordinators responded that costs related to MDSS were not estimated at the regional level.

II. OUTER SETTING			
A	Needs & Resources of those served by the organization: Extent to which the needs of WRA, especially for a safe and healthy birth, as well as barriers and facilitators, to meet those needs, are known and prioritized by the regional/provincial level.	--	Not applicable: the community has no role in reviews and response according to the national policy; No statements related to this construct
**B**	**Extra-organizational communication:**<br>Level of connectedness and networks with other health system levels, external actors and community	Inclusion Criteria: Statements about external relationship and communication (strong or weak, formal or informal, tangible or intangible), including community links, private and military health sector, university hospital, MOH, other regions, local authorities, partners as WHO, UNFPA, UNICEF...<br>Exclusion Criteria: Statements about internal communication and relationships, e.g descriptions of meetings, email groups, or other methods of keeping people connected and informed, and statements related to team formation, quality, and functioning (code to **Networks & Communications**).	
**C**	**Pressure:**<br>External actors pressure to implement MDSS	Inclusion Criteria: Statements about national and external subnational pressure to implement MDSS, political prioritization (national mobilization and awareness of issue), and perceptions around this pressure.<br>Exclusion Criteria: Statements about hierarchical pressure at district and regional level (code to **Leadership engagement**).	
**D**	**External policies & Incentives:**<br>External strategies to spread MDSS, including national policy and regulations, recommendations and guidelines, financial and non-financial rewards, and public or benchmark reporting.	Inclusion Criteria: Statements about national MDSS policy, death notification requirements, legal framework, litigation/legal protection, technical guidance, confidentiality;<br>Descriptions of external performance measures e.g. national confidential enquiry reports, media, performance reports;<br>Statements about incentives systems (monetary and others) that are controlled outside the organization.<br>Exclusion Criteria: Statements related to incentive systems set up on subnational level initiative by the health region directorate or the district (code to **Leadership engagement**).	

III. INNER SETTING			
**A**	**Structural Characteristics:**<br>Team composition and characteristics including who comprises the team.	Inclusion Criteria: Statements about team size, interdisciplinary nature, staff turnover, internal division of labor, competing priorities, level of staff interest, nature of meetings (e.g., hierarchical), centralization (the concentration of decision-making autonomy).<br>Exclusion Criteria: No criteria	
**B**	**Communications:**<br>Nature and quality of internal connections between individuals (including hierarchies, mentorship, teamwork, and management).	Inclusion Criteria: Statements about team relationship, the nature of internal communication, hierarchies within teams, strategies such as mentorship, collaboration, support from managers, management and networking within and across teams and among stakeholders involved in the implementation process, teamwork approach i.e. involving consensus building, inclusiveness, and delegation of responsibility.<br>Exclusion Criteria: Statements about networking and communication outside the organization (code to **Extra-organizational communication**); Statements related to implementation leaders' and users' access to information and training on MDSS (code to **Access to Knowledge & Information**); Statements related to engagement strategies and outcomes, e.g., how key stakeholders became engaged with the MDSS implementation and what their role is (code to **Engaging**).	
**C**	**Cultural environment:**<br>Cultural factors that contribute to a positive or negative environment for MDSS implementation.	Inclusion Criteria: Statements about (non) supportive culture at a personal, institutional, provincial, and regional level, fostering of professionalism, an ethos of safety e.g. culture of accountability, blame culture and punitive measures vs trust, learning, and improvement.<br>Exclusion Criteria: Statements about learning climate (code to **Implementation Climate**).	
**D**	**Implementation Climate:**<br>The absorptive capacity for change shared receptivity of involved individuals in the MDSS, and the extent to which MDSS implementation will be expected, rewarded, supported, within the region/district.	Six sub-constructs contribute to the implementation climate for an intervention: Tension for Change, Compatibility, Relative Priority, Organizational Incentives and Rewards, Goals and Feedback, and Learning Climate.	
1	*Tension for Change:*<br>Degree to which stakeholders perceive the current status of maternal mortality as intolerable or needing action.	Inclusion Criteria: Statements that (do not) demonstrate a strong need for the MDSS and/or preventable maternal deaths as intolerable, e.g., statements that the MDSS is absolutely necessary or redundant with other programs.<br>Exclusion Criteria: Statements that demonstrate the MDSS is better (or worse) than existing programs (code to **Relative Advantage**).	
2	*Compatibility:*<br>Degree of tangible fit between meaning and values attached to the MDSS by involved individuals, how those align with individuals' own norms, values, and perceived risks and needs, and how the MDSS fits with existing workflows and systems.	Inclusion Criteria: Statements that demonstrate the level of MDSS compatibility with organizational values and work processes; that the MDSS did (or did not) need to be adapted as evidence of compatibility or lack of compatibility<br>Exclusion Criteria: Statements regarding the flexibility and (in)ability to adapt and change the MDSS to regional/provincial context, operating conditions, and information needs, depending on workflows of individual organizations, e.g., complaints about the rigidity of processes, variations in implementation observed across districts, suggestions for MDSS improvement (code to **Adaptability**).	
3	*Relative Priority:*<br>Individuals' shared perception of the importance of the implementation within the organization.	Inclusion Criteria: Statements that reflect the relative priority of the MDSS, e.g., statements related to change fatigue in the organization due to implementation of many other programs.<br>Exclusion Criteria: Statements regarding the MDSS priority based on compatibility with organizational values, e.g., if MDSS is not prioritized because it is not compatible with organizational values (code to **Compatibility)**.	

4	*Organizational Incentives & Rewards:*<br>Extrinsic incentives such as goal-sharing awards, performance reviews, promotions, per diems, training, and less tangible incentives such as increased stature or respect.	Inclusion Criteria: Statements related to whether incentive systems are in place to foster (or hinder) implementation, e.g., rewards or disincentives for staff engaging in the MDSS (extra training, financial motivation, per diems, transportation), or removal of funding that financed incentives.<br>Exclusion Criteria: Statements about incentives systems that are controlled outside the organization (code to **External policies & Incentives**).	
5	*Goals and Feedback:*<br>The degree to which goals are clearly communicated, acted upon, and fed back to staff, and alignment of that feedback with goals. It includes organizational processes and supporting structures independent of the implementation process.	Inclusion Criteria: Statements related to MDSS objectives, (lack of) its alignment with larger organizational goals, as well as feedback to staff regarding those goals, e.g., regular audit and feedback regarding the gap between the current organizational status and the future (goal) organizational status.<br>Exclusion Criteria: Statements that refer to the (lack of) implementation team's assessment of the progress toward and impact of implementation, as well as the interpretation of outcomes related to implementation (code to **Executing, Reflecting & Evaluating**).	
6	*Learning Climate:*<br>A climate in which leaders express their own fallibility and their need for team members' assistance and input; team members feel that they are essential, valued, and knowledgeable partners in the change process; individuals feel psychologically safe to try new methods; and there is sufficient time and space for reflective thinking & evaluation.	Inclusion Criteria: Statements that support (or refute) the degree to which key components of the organization exhibit a "learning climate."<br>Exclusion Criteria: Statements about (non) supportive culture, fostering of professionalism, an ethos of safety e.g. culture of accountability, blame culture and punitive measures vs trust, learning, and improvement (code to C**ultural Environment**).	
**E**	**Readiness for Implementation:**<br>Tangible indicators of organizational commitment to its decision to implement the MDSS, including committees formed, training programs, coordinators identified, leadership informed, availability of tools, HRH workload.	Readiness for implementation consists of three sub-constructs: Leadership Engagement, Available Resources, and Access to Knowledge and Information.	
1	*Leadership Engagement*:<br>Commitment, involvement, and accountability of leaders and managers with the implementation.	Inclusion Criteria: Statements regarding the level of engagement of organizational leadership (executive leaders, middle management, front-line supervisors, and team leaders, who have a direct or indirect influence on the implementation).<br>Exclusion Criteria: Statements regarding an organizational leader, e.g., a director takes the lead in implementing MDSS (code to **Engaging).**	
2	*Available Resources:*<br>Funding or resource support dedicated for MDSS implementation and on-going activities (e.g. sponsors, budgets)	Inclusion Criteria: Statements related to the presence or absence of resources specific to the MDSS.<br>Exclusion Criteria: Statements related to training (code to Access to **Knowledge and Information)**; Statements related to the quality of materials (code to **Complexity and Design Quality)**.	
3	*Access to Knowledge & Information:*<br>Ease of access to digestible information and knowledge about the MDSS and how to incorporate it into work tasks.	Inclusion Criteria: Statements related to implementation leaders' and users' access to knowledge and information regarding using the MDSS, i.e., experts, other experienced staff (including those not necessarily achieving "expert" status), training (effectiveness), documentation, supportive information, and computerized information systems.<br>Exclusion Criteria: Statements related to engagement strategies and outcomes, e.g., how key stakeholders became engaged with the MDSS and what their role is in implementation (code to **Engaging)**; Statements about communication, and relationships in the organization, (code to C**ommunications)**.	

<table>
<tr><th colspan="4">IV. INTERVENTION PROCESS</th></tr>
<tr><td>A</td><td>Planning:
The degree to which a scheme or method of behavior and tasks for implementing an intervention are developed in advance, and the quality of those schemes or methods.</td><td>--</td><td>Removed as the study was conducted 7-years after MDSS implementation.</td></tr>
<tr><td rowspan="2">B</td><td rowspan="2">Engaging:
Attracting and involving appropriate individuals in the implementation and use of the intervention through a combined strategy of social marketing, education, role modeling, training, supportive supervision, and other similar activities.</td><td><u>Inclusion Criteria:</u> Statements related to early (or late) engagement strategies and outcomes to help people feel ownership of the MDSS, i.e., if and how staff and innovation participants became engaged with the MDSS and what their role is in implementation, e.g Opinions Leaders, Formally Appointed Internal Implementation Leader, Champions, External Change Agents and other Key Stakeholders directly impacted by MDSS implementation.</td><td rowspan="2"></td></tr>
<tr><td><u>Exclusion Criteria</u>: Statements related to implementation leaders' and users' access to Knowledge and Information regarding using the program; about Communications in the organization; regarding Leadership Engagement if an implementation leader, champion, is also an organizational leader.</td></tr>
<tr><td rowspan="2">C</td><td rowspan="2">Executing, Reflecting & Evaluating:
Carrying out or accomplishing the MDSS implementation according to plan, and feedback about the progress and quality of MDSS components implementation accompanied with regular personal and team debriefing about progress, experience, and implementation outcomes.</td><td><u>Inclusion Criteria</u>: Statements that describe steps and components of MDSS implemented at the subnational level and demonstrate how implementation occurred with respect (or not) to the national MDSS framework;
Statements that refer to the implementation team's (lack of) assessment of the progress (or barriers) toward and impact of MDSS implementation, as well as the interpretation of outcomes related to implementation (data completeness, data quality, data use);
Statements about the state and modalities, when the implementation team discusses feedback in terms of adjustments needed to complete, improve, and sustain implementation (provincial quarterly reports, data management, and storage, performance indicators).</td><td rowspan="2">We decided to merge Executing and Reflecting & Evaluating because it was very difficult to separate these two constructs in analyzing the MDSS components and steps.</td></tr>
<tr><td><u>Exclusion Criteria:</u> Statements related to the (lack of) alignment of implementation and MDSS goals with larger organizational goals, as well as feedback to staff regarding those goals, e.g., regular audit and feedback showing any gaps between the current organizational status and the goal (code to Goals & Feedback);
Evidence of the integration of evaluation components used as part of "Reflecting and Evaluating" into on-going or sustained organizational structures and processes (code to Goals and Feedback);
Statements that capture reflecting and evaluating that participants may do during the interview, for example, related to the success of the implementation (code to Knowledge & Beliefs).</td></tr>
<tr><td>1</td><td colspan="2">Identify and investigate deaths among WRA:
Identification and investigation of WRA deaths on an ongoing basis in health facilities (hospitals, primary health care, transfer) and community, followed by immediate preliminary enquiry to identify pregnancy related deaths.</td><td></td></tr>
<tr><td>2</td><td colspan="2">Collect data:
Investigation of suspected maternal deaths in facilities and communities</td><td></td></tr>
<tr><td>3</td><td colspan="2">Maternal death review (MDR):
Review of maternal deaths by RC-MDRs i.e., Examination of medical and non-medical contributing factors that led to the death, assessment of avoidability and development of recommendations for preventing future deaths.</td><td></td></tr>
<tr><td>4</td><td colspan="2">Analyze and make recommendations:
Analysis and interpretation of aggregated findings from provincial reports and regional reviews: Reviews and priority recommendations for regional action are made based on the aggregated data at the regional level and reported to the national level.</td><td></td></tr>
<tr><td>5</td><td colspan="2">Respond and monitor response:
Implement recommendations made by the RC-MDRs and those based on aggregated data analyses. Actions can address problems at the community, facility or multisectoral level. Monitor and ensure that the recommended actions are being adequately implemented.</td><td></td></tr>
</table>
