# Supplementary figures and images for: Eight-year experience of maternal death surveillance in Morocco: qualitative study of stakeholders’ views at a subnational level
Source: BMC Public Health. 2022 Nov 18;22:2111. doi: 10.1186/s12889-022-14556-0 (PMC9673401; doi:10.1186/s12889-022-14556-0)

## Additional file 5: Summary of the node tree developed for thematic analysis (NVivo Starter 11).

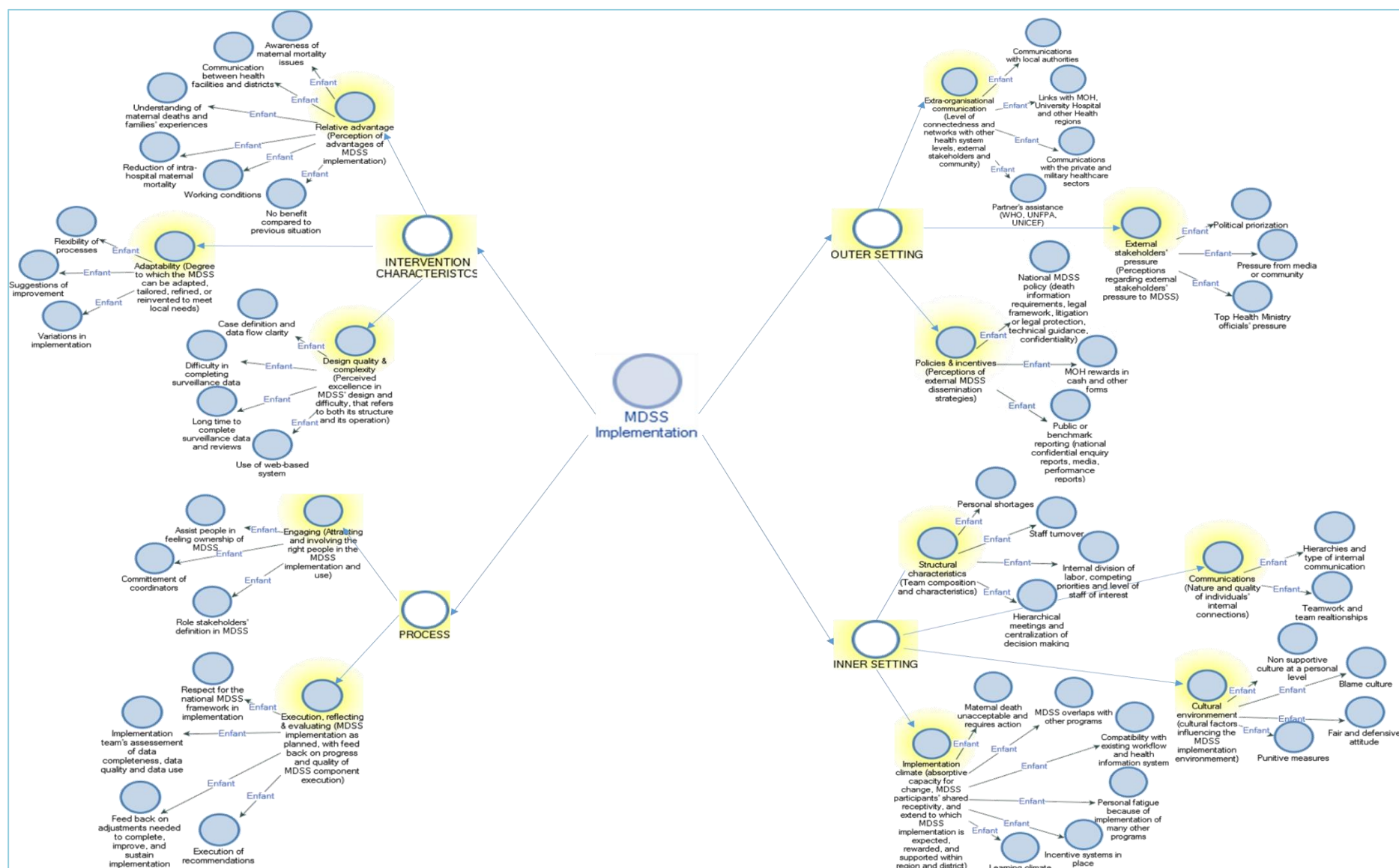

Supplement: Supplementary file 5 — Additional file 5. Summary of the node tree developed for thematic analysis (NVivo Starter 11). [file 12889_2022_14556_MOESM5_ESM.pdf]
