# Supplementary material for: Eight-year experience of maternal death surveillance in Morocco: qualitative study of stakeholders’ views at a subnational level
Source: BMC Public Health. 2022 Nov 18;22:2111. doi: 10.1186/s12889-022-14556-0 (PMC9673401; doi:10.1186/s12889-022-14556-0)
Supplement: Supplementary file 6 — Additional file 6. COREQ 32 item Checklist. [file 12889_2022_14556_MOESM6_ESM.pdf]

Additional file 6: COREQ 32 item Checklist

N°	Item	Comment	Reported on page number or not applicable (N/A)
**Domain 1: Research team and reflexivity**			
Personal Characteristics			
1.	Interviewer/facilitator	The PI (SA) conducted the IDIs	**7**
2.	Credentials	SA is M.D, M.P.H, PhD in Public Health Sciences<br>IG, WHZ and VDB are PhD in Public Health.	**N/A**
3.	Occupation	Researcher's occupations at the time of the study included research fellow and research professors.	**N/A**
4.	Gender	Three female-identifying (including the PI) and one male-identifying researchers	**N/A**
5.	Experience and training	Research team members have expertise in relevant areas: SA was trained to conduct interviews and analyze data of qualitative research and had a long experience in maternal deaths surveillance/reviews.<br>IG, WHZ and VDB are professors who had extensive experience of conducting qualitative and mixed research, on maternal health.	**N/A**
Relationship with participants			
6.	Relationship established	The position of SA as the head Morocco's National Unit of Maternal Mortality Surveillance from 2016 to 2018 (i.e., at the time of conducting the research) was not a hierarchical one. She was officially assigned responsibility for monitoring MDSS activities at national level. This position was extremely helpful in accessing the data and obtaining authorization to conduct interviews. Interviews were conducted with regional and district-level actors whose names were drawn up health-region officials. While SA had regular professional relationships with RCOs, for most participants, the interviews were their first contact with the PI. She gave no incentives to compensate for routine activities. To minimize descriptive and interpretive biases, regular feedback, and discussion of the findings in iterative processes were engaged with the Ph.D. supervisor (VDB) involving continuous questioning of the understanding of data and reviewing of findings.	**24,26**
7.	Participant knowledge of the interviewer	The interviewer introduced herself to participants as well as describing the purpose of the study and answered any questions participants may have had about the study and those involved in it.	**31**
8.	Interviewer characteristics	SA was aware of the national health system and MDSS in Morocco. The MDSS was the topic of her PhD thesis.	**26,27**
**Domain 2: study design**			
Theoretical framework			
9.	Methodological orientation and Theory	The research strategy of this study is based on a descriptive qualitative methodology.	**4**
Participant selection			
10.	Sampling	Purposive sampling was used (Health region-level stakeholders who contribute to the maternal death surveillance and/or review process, use the results obtained, or have influence over the MDSS's implementation and sustainability due to their position)	**6**
11.	Method of approach	IDIs, Face-to-face	**7**
12.	Sample size	Among 47 potential participants, 31 were interviewed.	**6**
13.	Non-participation	We could not find contact information for eight health workers (retirement, departure for private practice, or	**6**

		transfer to another region). One AN-RE refused to participate because he no longer wanted to be a RC-MDR member. Seven participants (one RHD, three OB-GYNs, one of whom left for private sector, two AN-REs, and one midwife) were not available, even after callbacks.	
Setting			
14.	Setting of data collection	Except, three participants (a RHD, an OB-GYN, and a head of RHO) that were interviewed via phone, interviews took place in health region office.	**7**
15.	Presence of non-participants	None; the IDIs were conducted in a private location at the health region office.	**7**
16.	Description of sample	Characteristics of the participants are presented in Table 2.	**11**
Data collection			
17.	Interview guide	The interview guide was open but listed several key questions. The PI organized interviews with similar representatives of RC-MDR in one region (not included in the study) to test the interview guide prior to use. Some questions were adjusted or added considering data obtained from earlier interviews in the study regions.	**7**<br>**Additional file 2**
18.	Repeat interviews	Interviews were not repeated.	**N/A**
19.	Audio/visual recording	We used audio recording to collect the data. Except for four participants who refused to be recorded, all interviews were audio-recorded and later transcribed. Two student midwives were recruited and paid as freelancers to transcribe the interviews at the PI's office under her supervision.	**8,26**
20.	Field notes	The interviewer wrote brief field notes after each interview regarding the nature of the interview and her perceptions of the participants responses.	**N/A**
21.	Duration	Interview times varied but the mean length of interviews was around 67 minutes.	**10**
22.	Data saturation	Data saturation was discussed from an individual-oriented perspective. Each interview was considered complete when no new information on the MDSS system seemed to emerge and the interviewer had a full understanding of the participant's perspective. .	**8,9**
23.	Transcripts returned	No	**N/A**
**Domain 3: analysis and findings**			
Data analysis			
24.	Number of data coders	SA coded the data and described codes, and these were discussed with VDB on a regular basis.	**9**
25.	Description of the coding tree	The research team has developed and refined a codebook to describe the selected as well as the rejected or merged constructs and the underlying justification.	**9,10**<br>**Additional file 5**
26.	Derivation of themes	The themes were those used in the CFIR.	**9**
27.	Software	SA completed the initial coding in NVivo Starter 11. The results were exported to a word document and the content of each code was reviewed with VDB to check if the content was correct and if some categories should be combined.	**8**
28.	Participant checking	No	**N/A**
Reporting			
29.	Quotations presented	Quotations that best illustrated the significant points shared by most respondents were selected and translated into English by SA. The translation was discussed with co-authors and revised by a professional translator. Quotations are presented and identified in a manner protecting participants confidentiality.	**10**

30.	Data and findings consistent	Data reliability was confirmed by comparing the encoding produced independently. Agreements and discrepancies were documented and any divergence was resolved by consensus, through discussion within the research team. A codebook was developed and refined to describe both the selected and rejected or merged constructs as well as the underlying justification. The adapted CFIR was used toguide data coding, data analysis and findings reporting.	**9**<br>**Additional file 4**
31.	Clarity of major themes	Major themes are clearly identified.	**12-19**<br>**Additional files 4, 5**
32.	Clarity of minor themes	Minor themes (CIFR constructs) are clearly identified and related to major themes.	**12-19**<br>**Additional files 4, 5**

2
