# Supplementary material for: Eight-year experience of maternal death surveillance in Morocco: qualitative study of stakeholders’ views at a subnational level
Source: BMC Public Health. 2022 Nov 18;22:2111. doi: 10.1186/s12889-022-14556-0 (PMC9673401; doi:10.1186/s12889-022-14556-0)
Supplement: Supplementary file 7 — Additional file 7. Grey literature. [file 12889_2022_14556_MOESM7_ESM.pdf]

Additional file 7: Grey literature

- Comité national d'experts sur l'audit confidentiel des décès maternels (CNEAC). Rapport national de l'enquête confidentielle des décès maternels de 2009. Rabat, Maroc: Ministère de la Santé; 2010.
- Comité national d'experts sur l'audit confidentiel des décès maternels (CNEAC). Rapport national de l'enquête confidentielle des décès maternels de 2010. Rabat, Maroc: Ministère de la Santé; 2013.
- Comité national d'experts sur l'audit confidentiel des décès maternels (CNEAC). Rapport national de l'enquête confidentielle des décès maternels de 2015 [Internet]. Rabat, Maroc: Ministère de la Santé; 2017. https://www.sante.gov.ma/Publications/Documents/rapport%20SSDM%20final% 20 Edit% C3%A9%20d%C3%A9c%202015%20.pdf
- Darkaoui K, Abouchadi S, Khassouani C, Kruk ME, El Adawy M, Belghiti Alaoui A, et al. Réduire la mortalité maternelle au Maroc : Partager l'expérience et soutenir le progrès. Rabat, Maroc: Ministère de la Santé; 2011. http://www.abhatoo.net.ma/maalama-textuelle/developpement-durable/societe-durable/sante-humaine/maladies-liees-a-l-environnement/maladies-du-systeme-immunitaire/reduire-la-mortalite-maternelle-au-maroc-partager-l-experience-et-soutenir-le-progres
- Direction régionale de la santé de Souss Massa Draa. Rapport régional des enquêtes confidentielles autour des décès maternels de la région de Souss Massa Draa 2015. Agadir, Maroc: DRS Souss Massa Draa; 2017.
- Direction régionale de la santé de Tadla-Azilal. Premier Rapport Régional Sur les Décès Maternels au titre de l'année 2015. Béni Mellal, Maroc: DRS Tadla Azilal; 2017.
- Institut National d'Administration Sanitaire (INAS), FNUAP, AMDD, Colombia University. Atelier National de consensus : Les audits des soins obstétricaux. Rabat, Maroc : INAS et AMDD, Colombia University; 2001.
- Ministère de la Santé [Maroc]. Plan d'action 2008 – 2012 pour accélérer la réduction de la mortalité maternelle et infantile. Rabat : Ministère de la Santé ; 2008. http://www.abhatoo.net.ma/maalama-textuelle/developpement-durable/societe-durable/sante-humaine/nutrition-et-soins-de-sante/traitements-medicaux/plan-d-action-pour-accelerer-la-reduction-de-la-mortalite-maternelle-et-infantile-periode-2008-2012
- Ministère de la Santé [Maroc], Lettre N° 1268, 8 septembre 2008, Déclaration des décès de femmes de 15-49 ans.
- Ministère de l'Intérieur, dgcl/dpe/dhsp [Maroc], Lettre N° D6918, 29 septembre 2008, Déclaration obligatoire des décès de femmes de 15-49 ans.
- Ministère de la Santé [Maroc], Circulaire ministérielle n° 103, 3 décembre 2008, Notification des décès maternels.
- Ministère de la Santé [Maroc], Décision ministérielle n° 24, 6 janvier 2009, Désignation des membres du comité national d'experts d'audit confidentiel des décès maternels et néonatals (CNEAC).
- Ministère de la Santé [Maroc]. Modalités pratiques de mise en place du système de surveillance des décès maternels (guide). Rabat : Ministère de la Santé, DHSA ; 2009.
- Ministère de la Santé [Maroc], Circulaire ministérielle n° 118, 3 juin 2009, Lancement de l'enquête confidentielle des décès maternels.
- Ministère de la Santé [Maroc]. Plan d'action 2012 – 2016 pour accélérer la réduction de la mortalité maternelle et néonatale. Rabat : Ministère de la Santé ; 2012. http://www.abhatoo.net.ma/maalama-

textuelle/developpement-economique-et-social/developpement-social/sante/politique-sanitaire/plan-d-action-2012-2016-pour-acceler-la-reduction-de-la-mortalite-maternelle-et-neonatale-fin-du-compte-a-rebours-2015

- Ministère de la Santé & Ministère de l’Intérieur [Maroc], Circulaire conjointe n°69, 20 octobre 2015 (CR 4154/ 6 octobre 2015), Renforcement du système de surveillance des décès maternels.
- Ministère de la Santé [Maroc], Circulaire ministérielle n° 150, 27 avril 2016, Domiciliation du système de surveillance des décès maternels à l’Ecole Nationale de Santé Publique.
- Ministère de la Santé [Maroc], Eliminer les décès évitables des mères et des nouveau-nés, Stratégie 2017-2021, Janvier 2017.
- Ministère de la Santé [Maroc], Décision ministérielle n° 13867, 25 octobre 2017, Institution de Task Forces Régionales pour le pilotage des plans d’actions régionaux relatifs à la stratégie nationale d’élimination des décès évitables des mères, des nouveau nés et des enfants de moins de cinq ans.
- Ministère de la Santé [Maroc], Circulaire ministérielle n° 54, 5 novembre 2018, Domiciliation du système de surveillance des décès maternels, audits des décès néonatals et riposte à la Direction de la Population.
